# Supplementary material for: Exploring and Engineering Novel Strong Promoters for High-Level Protein Expression in Bacillus subtilis DB104 through Transcriptome Analysis
Source: Microorganisms. 2023 Dec 6;11(12):2929. doi: 10.3390/microorganisms11122929 (PMC10745405; doi:10.3390/microorganisms11122929)
Supplement: Supplementary file 1 [file microorganisms-11-02929-s001.zip › Supplementary Figure.pdf]

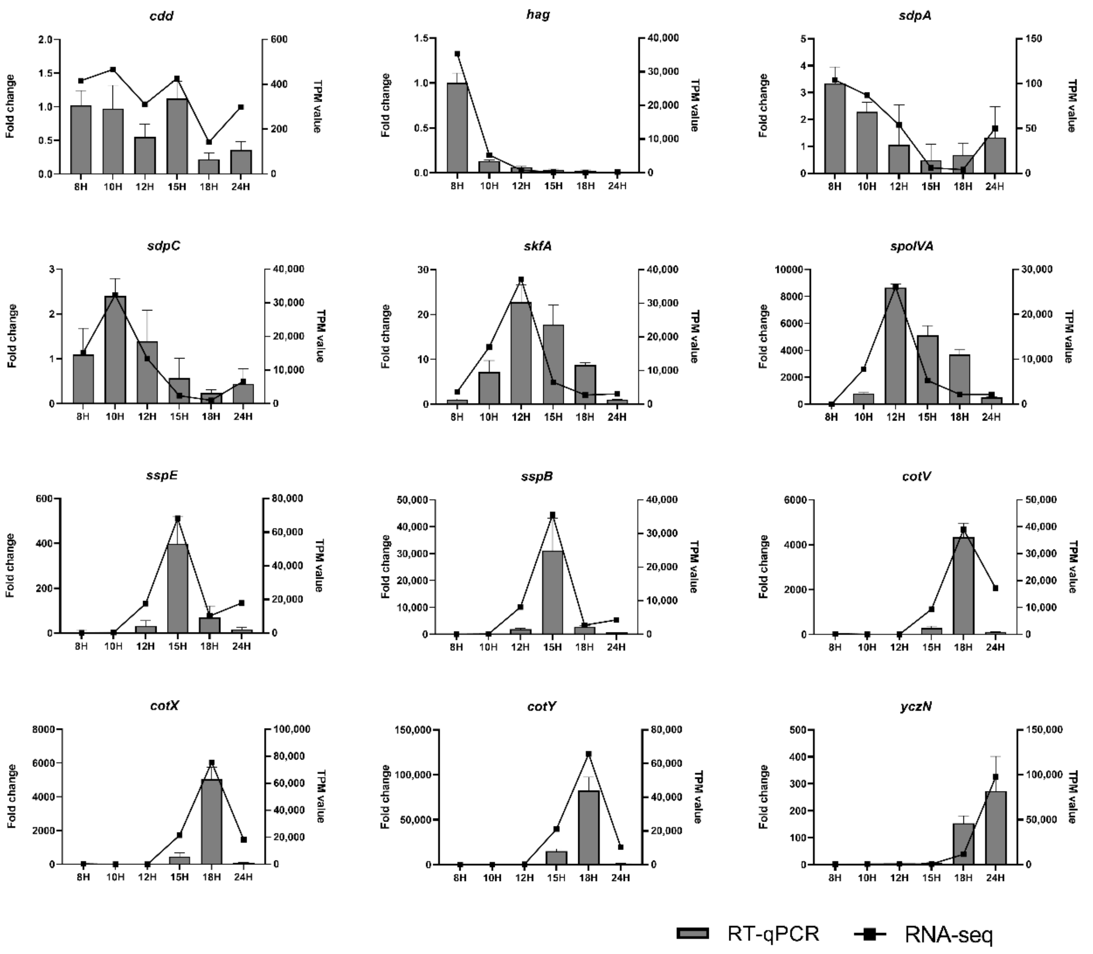

Figure S1. : Real-time quantitative reverse transcription PCR validation of genes transcribed by candidate promoters and control gene. The black square represents the TPM value of RNA-seq and the bar represents the fold change of RT-qPCR. RT-qPCR data are presented as the mean ± standard deviation (n = 3). The relative expression value was normalized to the *rpsJ* gene.

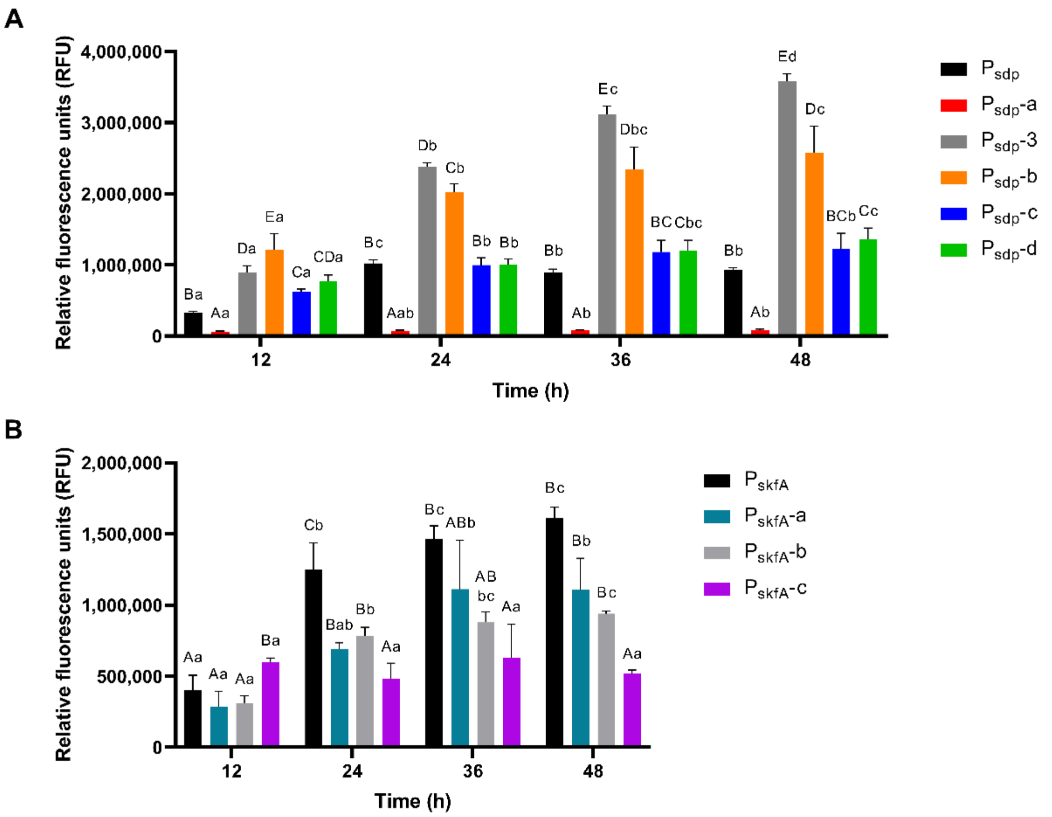

Figure S2. Engineering of P_{sdp} and P_{skfA} expression cassettes. **A** The eGFP expression level of strains containing P_{sdp} expression cassette derivatives. **B** The eGFP expression level of strains containing P_{skfA} expression cassette derivatives. Different lower-case letters denote significant differences between time within each recombinant strain, while different upper-case letters denote significant differences between expression cassettes at each time point ($P < 0.05$, ANOVA followed by Duncan's post hoc test).

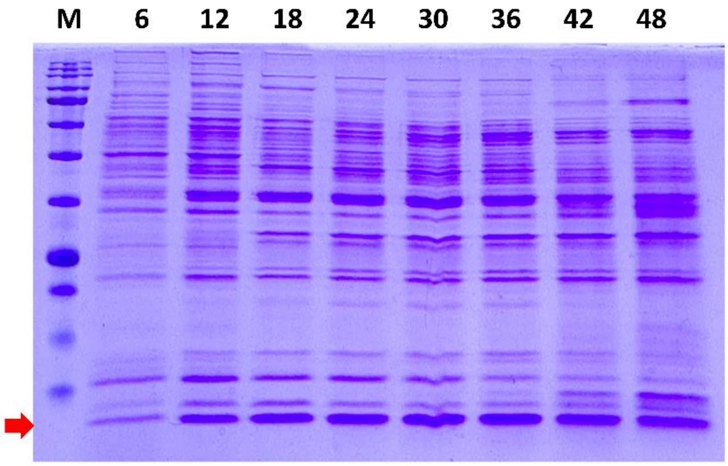

Figure S3. Time-course expression of His-hEGF driven by P_{sdp}-4. lane M, Protein molecular maker; The His-tagged hEGF bands were indicated by red arrows.

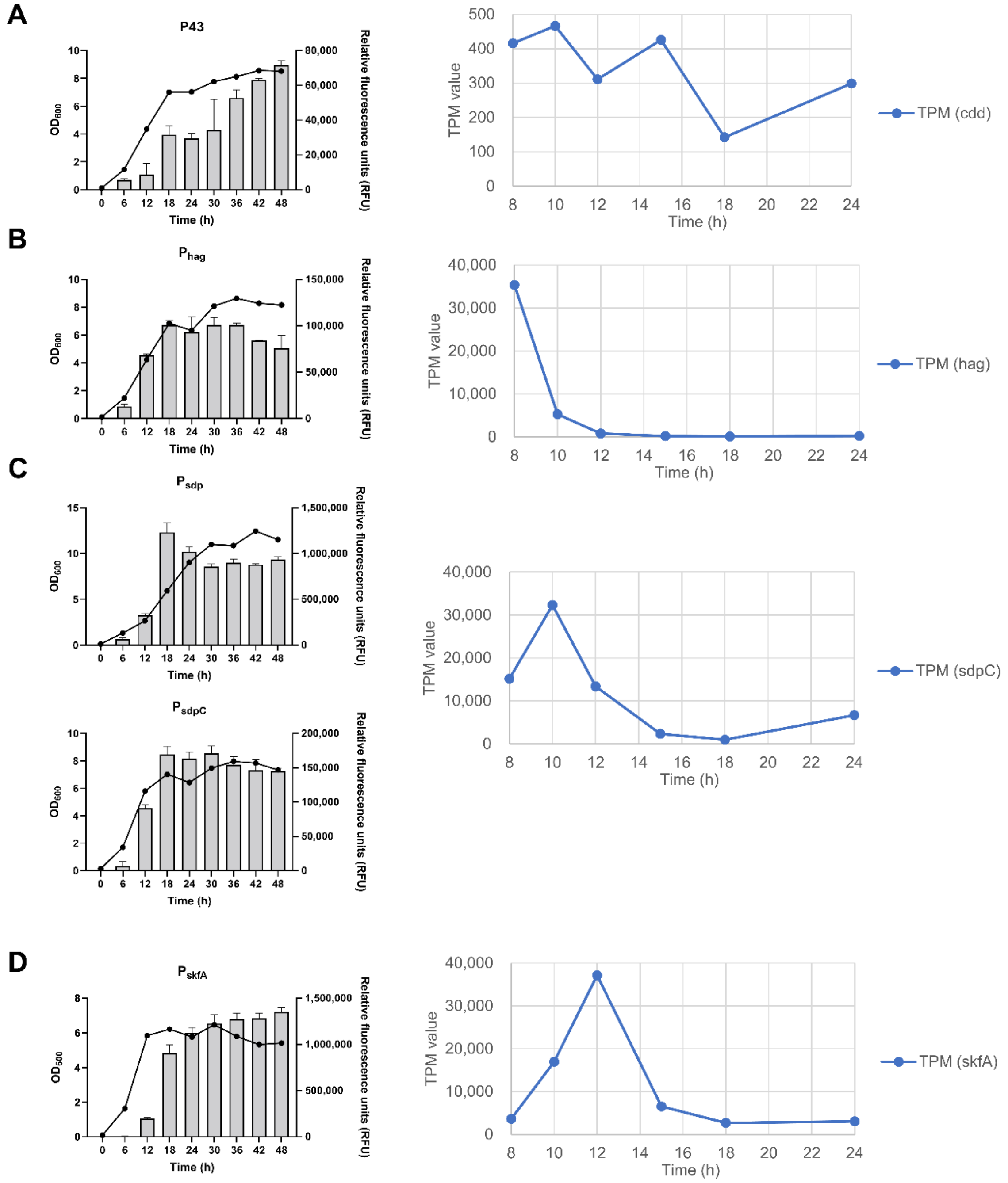

Figure S4. Cont.

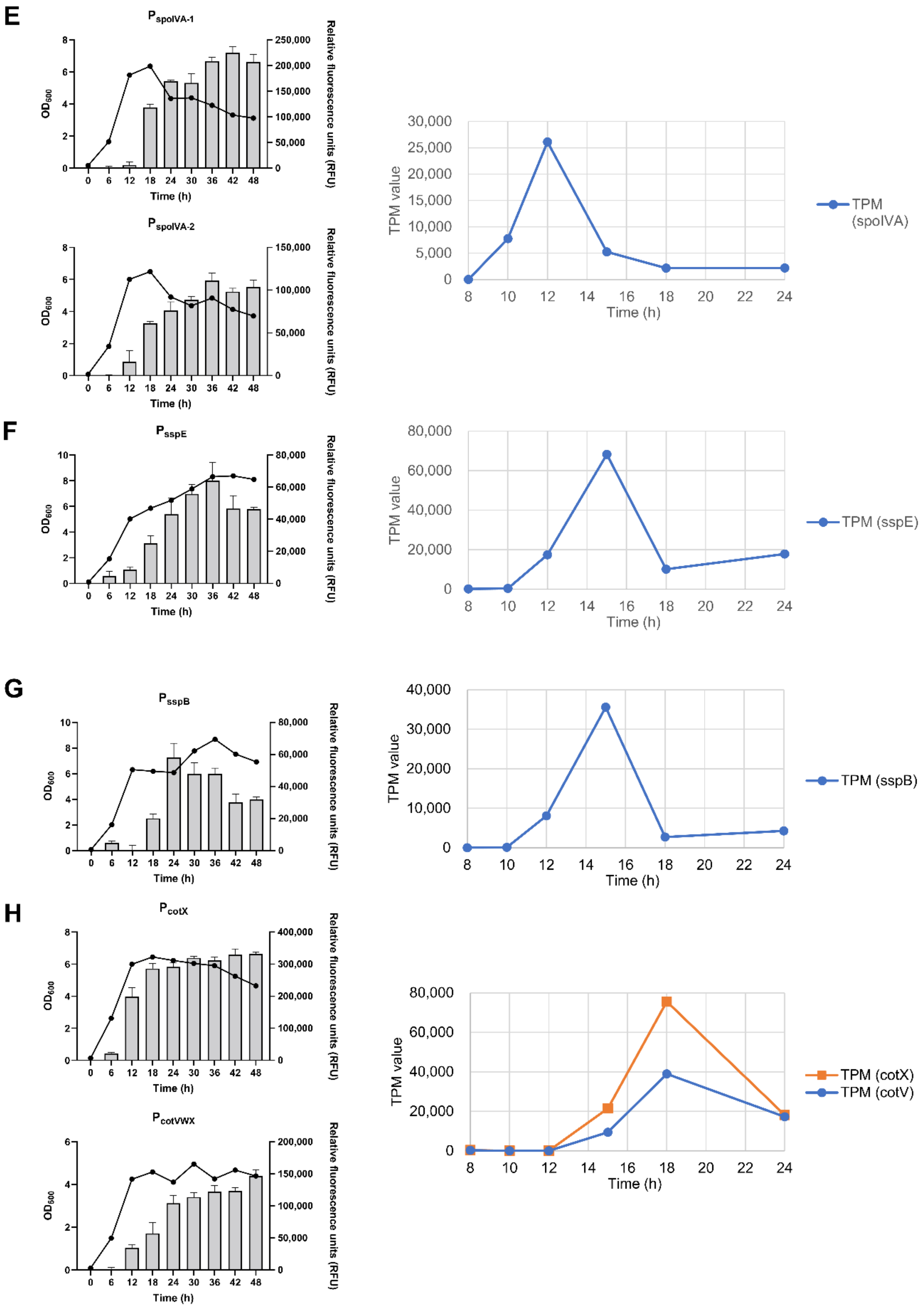

Figure S4. Cont.

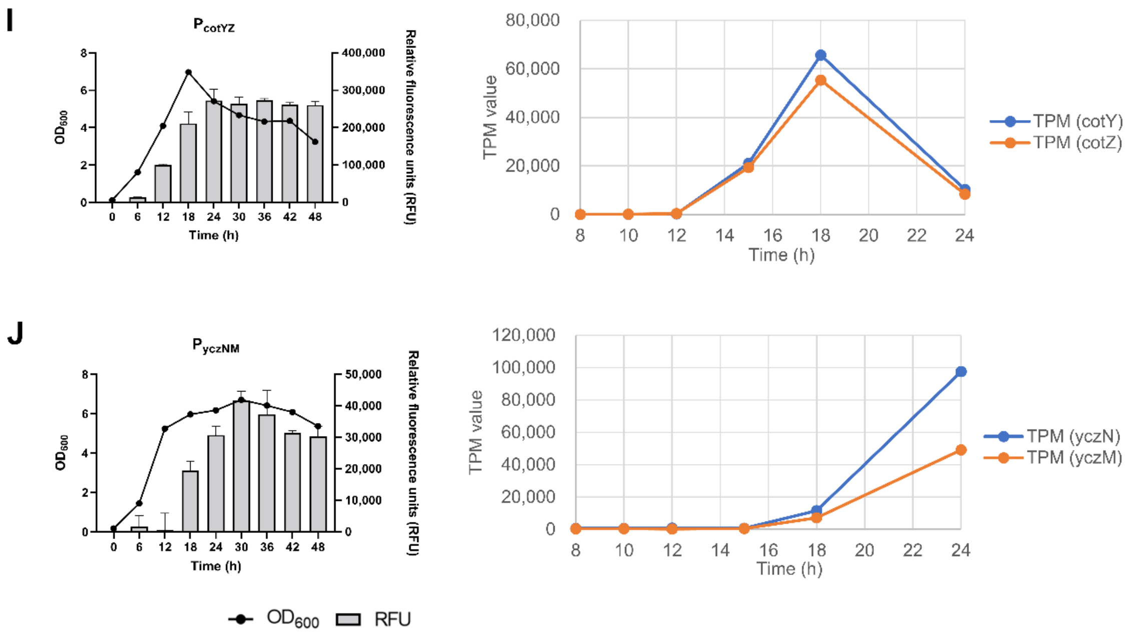

Figure S4. Expression patterns of eGFP and TPM value. The left panels are the eGFP expression patterns driven by **A** P43 **B** P_{hag} **C** P_{sdp} and P_{sdpC} **D** P_{skfA} **E** $P_{spoIVA\text{-}1}$ and $P_{spoIVA\text{-}2}$ **F** P_{sspB} **G** P_{sspE} **H** P_{cotX} and P_{cotVWX} **I** P_{cotYZ} and **J** P_{yczNM} and the right panel are the TPM value of their gene.

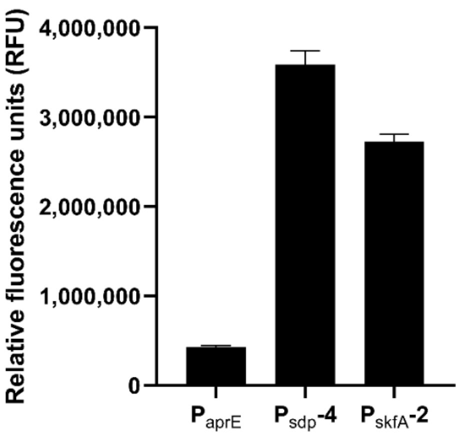

Figure S5. Maximum fluorescence intensities of P_{aprE}, P_{sdp}-4, and P_{skfA}-2.
